# Working memory improvements following supramaximal high-intensity interval training predicted by increased prefrontal cortex activation and leg strength gains

**DOI:** 10.1093/cercor/bhaf277

**Published:** 2025-10-14

**Authors:** Sofi Sandström, Emma Simonsson, Mattias Hedlund, Erik Rosendahl, Carl-Johan Boraxbekk

**Affiliations:** Department of Diagnostics and Intervention, Umeå University, 90185 Umeå, Sweden; Umeå Center for Functional Brain Imaging, Umeå University, Linnaeus väg 7, 90736, Umeå, Sweden; Umeå Center for Functional Brain Imaging, Umeå University, Linnaeus väg 7, 90736, Umeå, Sweden; Department of Community Medicine and Rehabilitation, Umeå University, Johan Bures väg 12, 90187 Umeå, Sweden; Department of Community Medicine and Rehabilitation, Umeå University, Johan Bures väg 12, 90187 Umeå, Sweden; Department of Community Medicine and Rehabilitation, Umeå University, Johan Bures väg 12, 90187 Umeå, Sweden; Department of Diagnostics and Intervention, Umeå University, 90185 Umeå, Sweden; Umeå Center for Functional Brain Imaging, Umeå University, Linnaeus väg 7, 90736, Umeå, Sweden; Institute for Clinical Medicine, Faculty of Medical and Health Sciences, University of Copenhagen, Blegdamsvej 3B 2200, Copenhagen, Denmark; Institute of Sports Medicine Copenhagen (ISMC) and Department of Neurology, Copenhagen University Hospital Bispebjerg, Bispebjerg Bakke 23, 2400, Copenhagen, Denmark

**Keywords:** FMRI, high-intensity training, older adults, prefrontal cortex, working memory

## Abstract

Physical exercise shows positive effects on cognitive functions such as working memory (WM) for older adults; however, large individual differences in response exist and the underlying mechanisms are not well understood. We tested the hypothesis that exercise-induced changes in cardiorespiratory fitness and leg strength would improve WM-related brain activity, which subsequently would improve WM performance. This study was based on the Umeå HIT study, a randomized controlled trial assessing the effects of watt-controlled supramaximal high-intensity interval training (HIT) versus moderate-intensity training for nonexercising older adults (*N* = 68). A subsample (*n* = 43, 66 to 79 years, 56% females) underwent task-based functional magnetic resonance imaging, testing WM. The outcomes of interest were change in WM performance, WM task activation, cardiorespiratory fitness, and leg strength. For WM performance, we found no significant between-group difference in change; however, there was a significant within-group increase for HIT in WM composites. For HIT, changes in leg strength significantly predicted increased right dorsolateral prefrontal cortex activation, which in turn predicted improved in-scanner WM task performance. Cardiorespiratory fitness did not predict WM-related functional change. These results indicate a specific physiological ingredient, namely leg strength gains, that is a potential mechanism in exercise-induced prefrontal activation and WM performance increases.

## Introduction

Healthy, nonpathological aging is associated with a decline in many cognitive functions, working memory (WM) being one among them (Nyberg et al. 2020). Cognitive decline is a predictor of mortality in older adults without dementia (Connors et al. 2015) and decreases in WM predict decline in instrumental activities of daily living such as managing medications and finances (Tomaszewski Farias et al. 2009; McDougall et al. 2019). Finding ways to maintain WM is of interest to prolong independent living.

WM tasks activate the medial prefrontal cortex (PFC) and dorsolateral PFC (dlPFC), along with lateral parietal and temporal areas, with increased task requirements, such as shifting from maintenance to manipulation, increasing frontal activation (Eriksson et al. 2015; Nyberg and Eriksson 2015). Longitudinal research has found aging to be associated with increases in bilateral parietal cortex activation during both maintenance and manipulation, as well as decreased recruitment of the left lateral PFC during manipulation related to decreased performance. This possibly indicates a failure to upregulate the PFC as task demands increase. The same study found that individuals who maintained stable left lateral PFC activation over time also maintained performance (Rieckmann et al. 2017). In this sense, maintaining a youthful pattern of neural activation is associated with stability in cognitive performance (Nyberg et al. 2012; Cabeza et al. 2018).

Support for physical exercise as an intervention mitigating age-related cognitive decline is mixed (Ciria et al. 2023), with many meta-analyses finding positive but modest effects (Northey et al. 2018; Falck et al. 2019) while also revealing large individual variability in response to exercise (Boa Sorte Silva et al. 2024). These differences are proposed to be in part due to differences in individual physiological responses to exercise, such as changes to one’s cardiorespiratory fitness, which in turn affect differences in neural change, and onward to change in cognitive functions (Voss and Jain 2022). For example, exercise-induced changes to cardiorespiratory fitness correlate with positive effects on WM performance (Frost et al. 2021), highlighting one potential physiological response necessary for cognitive gains. Furthermore, resistance training (RT) has been found to have a particular effect on WM (Northey et al. 2018). One study found that 24 weeks of either moderate- or high-intensity RT led to benefits in several WM tasks, compared to stretching and toning control (Cassilhas et al. 2007), indicating that metabolic demands different from those of aerobic exercise may induce WM adaptations. Thus, changes to muscular strength may be another potential active ingredient in the relationship between exercise and cognitive gains for older adults.

Meanwhile, the underlying mechanisms of physiological and neural changes following exercise are not well understood (Chen et al. 2020; Stillman et al. 2020). Most randomized controlled trials (RCTs) have used structural magnetic resonance imaging (MRI) or resting-state connectivity rather than task-based functional MRI (fMRI) despite suggestions that task-based imaging better captures cognitive aging in general (Ji et al. 2021; Mooraj et al. 2025). Some have found exercise-induced frontal upregulation, in particular in the middle frontal gyrus and superior frontal gyrus, with increased activation related to increased performance in executive functions (Wu et al. 2018) and increased cardiorespiratory fitness (Colcombe et al. 2004). In contrast, another study found frontal activation negatively correlated to change in cardiorespiratory fitness, interpreted as increased neural efficiency (Voelcker-Rehage et al. 2011). While these studies support exercise-induced neural changes to frontal areas, their differences in directionality highlight the need to further untangle patterns of task activation following exercise interventions, as well as a need to study other cognitive domains, such as WM. Understanding the underlying mechanisms of WM changes also encompasses a better understanding of the physiological changes necessary to induce neural and cognitive improvements, such as changes to cardiorespiratory fitness and muscle strength.

In an RCT comparing a supramaximal high-intensity interval training (HIT) protocol with a moderate-intensity training (MIT) protocol, we found a significant between-group difference in change for WM performance and isometric leg strength favoring supramaximal HIT. Irrespective of the group, there was a significant increase in peak oxygen uptake (VO_2_peak), despite the supramaximal HIT group exercising half the amount of time (Simonsson et al. 2023).

In this study, we examined the neural underpinnings of exercise-induced WM performance change, using an fMRI WM task. Based on the between-group differences in WM performance, we hypothesized a between-group difference in change in frontal activation during WM manipulation, with increased frontal activity in supramaximal HIT. Furthermore, we expected increases in prefrontal activation to be related to increases in isometric leg strength and WM performance for the HIT group only, while we expected cardiorespiratory fitness to drive change in frontal activation and WM performance for the whole group.

## Materials and methods

### Exercise intervention

The Umeå HIT study is an RCT assessing the effects of watt-controlled supramaximal HIT compared to MIT for older adults. The Regional Ethics Review Board in Umeå (2018-307-31M, 2018-421-32M) approved the study, and it is registered at ClinicalTrials.gov (NCT03765385). All the applicants provided written informed consent. A detailed description of the training intervention, as well as outcome variables not presented in this article, can be found in Simonsson et al. (2023).

In short, participants were recruited by advertisement in a local newspaper. The inclusion criteria were adults 65 years of age or older without engagement in regular moderate- or high-intensity exercise during the past year, and with the ability to take part in training twice weekly for 12 weeks. The exclusion criteria were heart or lung conditions with exercise-induced symptoms; movement-related dysfunction; insulin-treated diabetes; poor blood pressure control or untreated arterial hypertension; a mini-mental state examination score below 27; chronic or progressive neurological disease; and sharing a household with another study participant. All the included participants underwent extensive baseline testing, including measurements of cardiorespiratory, cardiovascular, metabolic, cognitive, and muscular function. A subsample of participants (*n* = 43) underwent brain MRI. After baseline testing, participants were randomized 1:1 to either supramaximal HIT or MIT, exercising on stationary bicycles twice weekly for 12 weeks, for a total of 25 sessions. The supramaximal HIT protocol (Hedlund et al. 2019; Simonsson et al. 2023) was 20 min in total, and included 10× 6-s intervals at supramaximal intensity, i.e. an external intensity in watts above that produced at an individual’s VO_2_peak. The MIT protocol was 40 min in total and included 3× 8-min bouts at a moderate intensity. In both exercise groups, the training intensity was individualized and calculated based on baseline testing. Follow-up testing was conducted within a week of the final exercise session.

### Participants

Power analysis for the Umeå HIT study indicated that a sample of 70 participants (estimated drop-out rate of 15%) was needed in order to detect between-group differences in change in the primary outcomes VO_2_peak (VO_2_peak, Mean Difference (MD) = 3.5 mL/kg/min, SD = 4.0) and global cognition [effect size (ES) = 0.79; Jonasson et al. 2017], with 80% power, and a two-sided *α* = 0.05. Comparable fMRI studies in the field have included a similar number of participants to that of our MRI subsample (Voelcker-Rehage et al. 2011; Wu et al. 2018; Prehn et al. 2019).

Of the 68 participants recruited to the Umeå HIT study, 43 (23 female) were selected to take part in MRI scanning. The MRI-scanning inclusion criterion was eyesight that could be corrected by scanner-compatible glasses, and the exclusion criterion was standard contraindications to MRI scanning, such as claustrophobia or metal implants. If neurological artifacts were found at MRI acquisition, participant data were excluded from analyses and no follow-up was conducted. MRI scanning was conducted on a test day separate from other testing. Prior to entering the MRI scanner participants repeated the alphabet, to ensure they had adequate literacy skills, and were then introduced to the task to ensure they understood the instructions, in line with previous studies on the same task paradigm (Pudas et al. 2009; Nyberg et al. 2014). The participant demographics can be found in Table 1.

**Table 1:** MRI sample vs non-MRI sample baseline characteristics.

	**MRI (*n* = 43)**	**Non-MRI (*n* = 25)**	***P***
**Group**			
** HIT, no. (%)**	22 (51.2)	12 (48.0)	0.80
**Demographics**			
** Women, no. (%)**	24 (55.8)	14 (56.0)	0.99
** Age, years**	69.77 (2.93)	69.52 (3.04)	0.74
** Education, years**	14.58 (3.03)	13.96 (3.75)	0.48
**Anthropometrics**			
** BMI, kg/m** ^**2**^	25.85 (3.25)	26.91 (4.92)	0.34
**Cognitive function**			
** Global cognition**	0.07 (0.91)	−0.12 (1.15)	0.50
** Offline WM composite**	−0.01 (1.06)	0.02 (0.91)	0.92
**Physiology**			
** VO**_**2**_ **peak, mL/kg/min**	23.72 (5.24)	21.51 (3.98)	0.06
** Isometric leg strength, N·m/kg**	1.74 (0.38)	1.61 (0.31)	0.15

Note: Numbers are presented as mean (SD) unless stated otherwise. A *t*-test was used to analyze differences in continuous variables and a chi-square test was used to analyze differences for categorical variables. BMI, body mass index.

### Cognitive testing

Global and domain-specific cognitive functions were assessed with a test battery consisting of 10 tests targeting 4 domains, including WM. For the global cognitive composite raw test scores were standardized to *z*-scores using the baseline means and SDs of the entire sample and averaged to form a unit-weighted composite. The test battery has previously been shown to be reliable (Pantzar et al. 2018). Of interest for this article, offline WM was assessed with two cognitive tests: the backward digit span (BDS) and the automated operation span (AOS). Both tests were computerized.

For BDS (digital version of WAIS-R) (Wechsler 1981), participants were presented with a sequence of numbers on the screen for 1 s each, with an interstimulus interval of 250 ms. The task was to memorize the sequence in its reverse order and then respond using the digit pad on the keyboard. The participant was given a practice round with a two-digit sequence, including feedback, after which the test began. Correct responses resulted in the sequence length increasing by 1 digit, to a maximum of 9 digits, while an incorrect response resulted in the same digit length being repeated. No feedback was given during the test, and the test ended after 2 incorrect responses at the same level. The dependent measure was the highest correctly completed sequence length.

For AOS (Unsworth et al. 2005), participants were presented with simple mathematical tasks and proposed solutions on the screen and were asked to indicate true or false if the solution was correct. Between each mathematical task, a letter was presented for 1 s. The task was to memorize the letter sequence presented while maintaining 85% accuracy on the mathematical tasks. After each block, participants were presented with 12 letter boxes on the screen and were to indicate the block’s letter sequence. A practice block, during which the participants practised each part of the task separately and then together, was given. The test itself consisted of 10 blocks of 3 to 7 letter sequences. The dependent measure was the sum of correctly remembered sets multiplied by respective set size.

For performance, two different composite scores were calculated. First, test scores on the AOS and BDS were standardized based on the whole group (both MRI and non-MRI participants, *n* = 68) means and SDs at baseline and an offline WM composite was calculated. Second, test scores on the AOS, BDS, and in-scanner WM manipulation were standardized based on the MRI subsample’s (*n* = 43) means and SDs at baseline, and an MRI WM composite was calculated.

### Fitness testing

VO_2_peak (mL/kg/min) was measured using a standardized ramp test on an electronically braked cycle ergometer (Monark 839E Monark Exercise AB, Vansbro, Sweden), and the outcome measure was the highest recorded oxygen uptake during the test. The exact parameters of the ramp protocol, including the termination criteria, can be found in Simonsson et al. (2023).

The maximal isometric leg strength (normalized joint torque in Newton meter per kilogram) was measured using a load cell (Lutron FG-5100, Lutron Electronic Enterprise Co., Ltd, Taipei, Taiwan) positioned in a Biodex dynamometer chair, with two trials per leg. The outcome measure was the maximal torque (N m) calculated from the highest force (N) of two trials multiplied by the moment arm (m) and then averaged across both legs and divided by weight.

### MRI acquisition

MRI data were acquired on a GE Discovery MR750 3T MRI scanner. Sequences of relevance for this study include an fMRI gradient-echo planar imaging sequence and a high-resolution *T*_1_-weighted structural image. The fMRI run lasted ~10 min and collected a total of 290 volumes (TR 2,000 ms; TE 30 ms; flip angle 80°; FOV 25 cm; 37 transaxial slices of 3.4 mm; 0.5 mm gap), including 10 initial dummy scans for the fMRI signal to reach equilibration. A 3D fast spoiled gradient echo sequence (180 slices, 1 mm thickness; TR 8.2 ms; TE 3.2 ms; flip angle 12°; FOV 25 cm × 25 cm) was used for the high-resolution *T*_1_-weighted structural image.

### In-scanner WM task

**Fig. 1:**
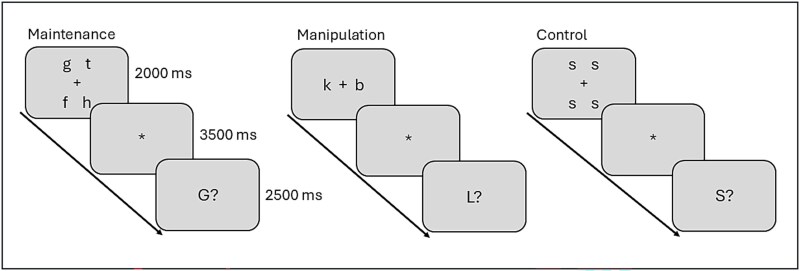
In-scanner WM task with three conditions: maintenance, manipulation, and control. Adapted from Pudas et al. 2009.

The blocked design in-scanner WM task consisted of three conditions: maintenance, manipulation, and control (see Fig. 1). There were six blocks of each condition, with three trials in each block. All the conditions followed the same timing parameters. For maintenance, the participant was shown four target letters for 2 s, followed by a 3.5-s presentation of a fixation cross, after which a probe letter was presented for 2.5 s, followed by another fixation cross for 0.5 s. During the presentation of the probe letter, the participant indicated with their right hand using a response pad whether the probe letter matched one of the four target letters. For manipulation, two target letters were shown and the participant indicated whether the probe letter was the subsequent letter in the alphabet to either target letter. For control, the four target letters were all the same, and the participant indicated whether the probe letter matched. Half of the probes were matches and the other half were nonmatches. All the target letters were lowercase, and all the probe letters were uppercase to minimize visual memorization. The performance outcome for in-scanner WM manipulation was the number of hits minus the number of false alarms for manipulation (Rieckmann et al. 2017). For stimulus presentation and recording of responses, E-Prime (Psychology Software Tools, Inc., Pittsburgh, PA) was used.

### Preprocessing and data analyses

FMRI raw data were preprocessed using an in-house Matlab-based (MathWorks, Inc., MA, United States) software (DataZ) utilizing SPM12 and included the following steps: slice-timing correction (interleaved order, first image set to reference slice); movement correction to unwarp and realign all scans to the first image of each volume; coregistration of the mean functional image and the structural *T*_1_ image; normalization of each scan from each time point to a sample-specific template based on baseline *T*_1_ images segmented into white matter, gray matter, and cerebrospinal fluid using DARTEL (Ashburner 2007); affine alignment to 2 × 2 × 2 Montreal Neurological Institute (MNI) standard space; and smoothing with an 8 mm Gaussian kernel. Final voxel size was 2x2x2 mm. Anatomical locations were determined from coordinates in MNI space (https://neurosynth.org/).

For first-level analyses, a general linear model was set up for each scan at each time point, and included the regressors for each condition, convolved with a canonical hemodynamic response function. Six realignment parameters as covariates of no interest were included. The contrast of interest was manipulation–maintenance.

For second-level analyses, a flexible factorial design model (Gläscher and Gitelman 2008) was fitted to examine between-group differences in change for the (manipulation–maintenance) contrast. As we were expecting small effects to due small differences between the exercise groups, all voxel-wise analyses were evaluated at *P <* 0.001, uncorrected, with an extension threshold of 10 voxels. To decrease the risk of type I errors, we limited the search area for all voxel-wise analyses by applying an explicit mask with a strict threshold (Family-wide error (FWE), *P <* 0.01) based on task-specific WM relevant brain regions for the same contrast (manipulation–maintenance) from a large sample of adults (*n* = 363) in the longitudinal Betula study using the same task as the present study (Nyberg et al. 2014). While this masking approach was intended to increase specificity, we emphasize that our analyses did not apply whole-brain correction for multiple comparisons and should therefore be considered exploratory, and the findings may serve to generate hypotheses for future studies with larger samples and more conservative statistical thresholds. All the analyses were performed in SPM12. Following the analysis of group differences, the first-level beta estimates for manipulation divided by the mean of session (MoS) were extracted and used to examine change–change associations with WM performance, VO_2_peak, and isometric leg strength.

To examine whether changes in isometric leg strength and VO_2_peak predicted changes to brain activity, we conducted two separate regression models. First, we used isometric leg strength change as a predictor for change in WM task activation for the supramaximal HIT group, with age, sex, and education years as further predictors. We also conducted a multiple linear regression to test whether change in VO_2_peak, age, sex, and education years in the whole group predicted change in WM task activation. For significant local maxima, we extracted the beta estimates using first-level estimates for manipulation divided by MoS. We then tested whether the beta estimates for significant local maxima were related to change in WM performance, again including age, sex, and education years as further predictors.

Performance on the offline WM composite, MRI WM composite, and in-scanner WM manipulation was assessed using linear mixed-effects models (LMMs), with the individual as a random effect, group and time as fixed effects, and age, sex, and education as covariates. We also used LMMs to analyze exercise-induced changes to VO_2_peak and isometric leg strength in the MRI sample, including age and sex as covariates.

R 4.4.1 (R Core Team 2020) and RStudio 2024.4.2.764 (RStudio Team, 2016) were used to analyze Pearson’s correlations, multiple regressions, and linear mixed models using the lme4 (Bates et al. 2015) and lmerTest (Kuznetsova et al. 2017) packages with two-tailed testing and *α* = 0.05.

## Results

### Participants

There were no significant differences between the MRI sample and those not selected for MRI with regard to age, sex, education years, BMI, global cognition, WM performance, or isometric leg strength at baseline (see Table 1). For VO_2_peak, the MRI sample had slightly higher baseline levels; however, this did not reach statistical significance.

### Exercise-induced changes to WM performance and fitness

**Fig. 2:**
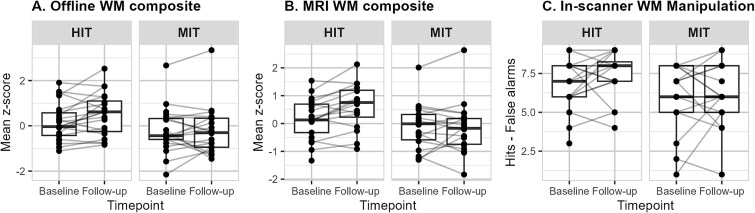
WM performance change. Group and individual change for supramaximal HIT and MIT. For the A) offline WM composite and B) MRI WM composite, means of standardized *z*-scores are reported. For C) in-scanner manipulation, raw scores (hits minus false alarms) are reported.

For the MRI sample, the between-group difference in change for the offline WM composite from Simonsson et al. (2023) was no longer significant, and neither was the MRI WM composite or the in-scanner WM task. There was a significant within-group increase for HIT in both the offline WM composite (*P* = 0.034) and the MRI WM composite (*P* = 0.008) (see Table 2 and Fig. 2). Further results on individual tasks can be found in the online supplementary material, see Table S1. For fitness measures, there was a significant between-group difference in change in isometric leg strength [MD = 0.08, 95% CI (0.00, 0.16)], with HIT showing a significant within-group change (*P* = 0.006). For cardiorespiratory fitness, there was no difference between the groups; however, similarly to Simonsson et al. (2023), both groups increased significantly with a mean increase of 1.49 mL/kg/min [95% CI (0.76, 2.21)], adjusted for sex and age. All the details can be found in Table 2.

**Table 2:** Within-group change and between-group differences in change from baseline to follow-up.

	**Within-group change**						**Between-group difference in change**			
	**HIT**			**MIT**			**Group × time**			
	**No.**	**Mean**	**SE**	**No.**	**Mean**	**SE**	**Mean**	**95% CI**	***P***	**ES**
**WM performance**										
Offline WM composite	22/20	0.29^*^	0.13	21/20	0.02	0.13	0.27	[−0.09, 0.64]	0.15	0.46
MRI WM composite	21/16	0.33^*^	0.12	18/20	0.02	0.12	0.31	[−0.00, 0.63]	0.07	0.66
In-scanner WM manipulation	21/16	0.39	0.39	21/20	0.06	0.36	0.33	[−0.69, 1.38]	0.53	0.21
**Fitness**										
VO_2_peak (mL/kg/min)	22/19	1.87^*^	0.52	21/20	1.14^*^	0.51	0.73	[−0.70, 2.14]	0.32	0.32
Isometric muscle strength (N m/kg)	22/20	0.08^*^	0.03	21/20	0.00	0.03	0.08	[0.00, 0.16]	0.06	0.62

Note: Values from linear mixed-effects models with the individual as a random effect. For analyses on WM performance, models are adjusted for age, sex, and years of education as fixed effects. For analyses on fitness models are adjusted for age and sex as fixed effects. Data are reported as LMM-estimated mean change with SE for within-group change, and 95% CI for between-group differences in change. ES was calculated from the model estimates of between-group difference in mean change and the unadjusted pooled standard deviation. HIT, supramaximal high-intensity interval training; MIT, moderate-intensity training; no., number of available measurements at baseline and follow-up presented as baseline/follow-up; CI, confidence interval; ES, effect size; VO_2_peak, peak oxygen uptake.

^*^Significant within-group change (*P* < 0.05) based on LMM estimates.

### Exercise-induced changes to WM activation

#### Between-group changes

In a flexible factorial model, we found a significant group × time interaction in the right putamen, with supramaximal HIT decreasing activity and MIT increasing activity (see Table 3). Irrespective of group we found a significant increase in activation in the right cerebellum.

**Table 3:** Areas with significant change in activation.

**Analysis**	**Location**				**No. of voxels**	**Ze**	***P***
	***X***	***Y***	***Z***	**Anatomical location**			
**Flexible factorial model**							
Group × time	24	20	2	Right putamen	10	3.22	<0.001
Main effect of time	38	−60	−38	Right cerebellum	27	3.67	<0.001
**Change-change analyses**							
* ▲*Strength as predictor (HIT group only)	48	24	40	Right dlPFC	32	3.89	<0.001

Note: All analyses uncorrected at *P* < 0.001. Anatomical locations as determined by coordinates in MNI space using https://neurosynth.org/. dlPFC, dorsolateral prefrontal cortex.

#### Associations to fitness and WM performance

Change–change correlation analyses found that change in activation in these areas was not significantly related to change in VO_2_peak [right putamen: Pearson’s *r*(33) = −0.02, *P* = 0.90; right cerebellum: *r*(33) = −0.09, *P* = 0.59], isometric leg strength [right putamen: *r*(34) = −0.05, *P* = 0.066; right cerebellum: *r*(34) = 0.10, *P* = 0.58], the offline WM composite [right putamen: *r*(34) = −0.21, *P* = 0.22; right cerebellum: *r*(34) = −0.32, *P* = 0.056], or the MRI WM composite [right putamen: *r*(31) = −0.15, *P* = 0.40; right cerebellum *r*(31) = −0.29, *P* = 0.096].

#### Change in fitness predicted changes in WM task activation

##### Isometric leg strength as fitness

**Fig. 3:**
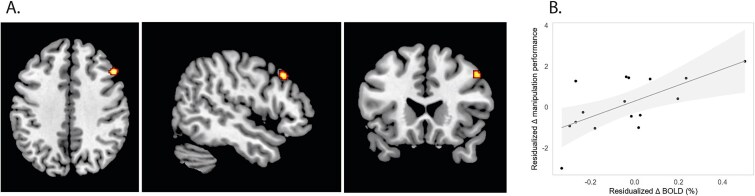
Leg strength gains predict right dlPFC functional change and performance increases. A) Change in leg strength positively predicting change in right dlPFC activation. Significant cluster is highlighted (yellow and red) and overlaid on ch2better.Nii.Gz template using MRIcron. B) Change in right dlPFC activation predicts in-scanner performance gains.

For HIT, change to isometric leg strength, predicted change in WM activation in the right dlPFC in a multiple regression including age, sex, and education years [*F*(4, 11) = 7.26, *P* = 0.005], explaining 63% of the variance in activation change (*r^2^*_adj_ = 0.63) (see Table 3). Among the predictors, change in isometric leg strength was a significant predictor of right dlPFC activation change [*β* = 0.87, SE = 0.32, *t*(11) = 2.67, *P* = 0.022]. However, age (*β* = 0.01, *P* = 0.43), sex (*β* = 0.13, *P* = 0.13), and education years (*β* = −0.01, *P* = 0.39) were not significant. In turn, right dlPFC activation change predicted in-scanner WM manipulation performance change in a multiple regression analysis with dlPFC activation change, age, sex, and education years as predictors (see Fig. 3). The overall model was statistically significant [*F*(4, 11) = 3.40, *P* = 0.05], explaining ~39% of the variance in performance change (*r^2^*_adj_ = 0.39). Among the predictors, change in right dlPFC activation was a significant predictor of in-scanner WM manipulation performance change [*β* = 4.08, SE = 1.36, *t*(11) = 3.00, *P* = 0.012]. However, age (*β* = 0.27, *P* = 0.09), sex (*β* = −0.51, *P* = 0.47), and education years (*β* = 0.02, *P* = 0.85) were not significant predictors.

##### VO_2_peak as fitness

We found that VO_2_peak change did not predict change to WM activation for the whole group.

## Discussion

This study investigated the neural underpinnings of exercise-induced WM increases observed following 12 weeks of supramaximal HIT. While no between-group differences to WM-related activation were found, the results indicated that improved isometric leg strength in the supramaximal HIT group was significantly related to increased brain activation in the right dlPFC during a WM task, which in turn predicted improved WM performance. Our findings propose a specific ingredient, gains to leg strength, that could be an important physiological target when designing exercise interventions intended for WM gains (Stillman et al. 2020; Voss and Jain 2022).

We identified leg strength as a physiological adaptation related to neural change. Previous findings connecting exercise-induced change between physiology, the brain, and cognition have primarily focused upon maximal oxygen uptake (VO_2_max) as a key ingredient (Voss and Jain 2022). For example, change to VO_2_max was positively associated with change in hippocampal volume, which in turn was associated with improvements to spatial memory (Erickson et al. 2011; Maass et al. 2015). Our findings suggest that leg strength gains may be another physiological target, this time predicting prefrontal activation changes following exercise. Previous studies have found an effect of RT on WM performance (Cassilhas et al. 2007; Northey et al. 2018), and while supramaximal HIT is not RT per se, exercise intensity in the supramaximal HIT program was above the individuals’ anaerobic thresholds, enabling adaptations beyond aerobic, such as a significant increase in isometric leg strength (Simonsson et al. 2023). Increases in strength during the first months of RT are primarily explained by peripheral and central neural adaptations (Kidgell et al. 2017). Based on the results of the present study, we propose that the gains in isometric leg strength related to prefrontal upregulation in the supramaximal HIT group may indicate a muscle–brain crosstalk enabling the adaptation. There are numerous potential muscle-releasing factors (myokines) that may mediate the muscle–brain crosstalk (Pedersen 2019). Circulating brain-derived neurotrophic factor is one example, which increases with exercise and is considered an indicator of brain health as it can cross the blood–brain barrier, affecting neuronal protection, remodeling, and survival, as well as synaptic plasticity (Gholami et al. 2024).

The right dlPFC has been implicated in many higher order processes, including WM (Eriksson et al. 2015). Previous research has found exercise-induced prefrontal upregulation connected to increased executive function performance (Wu et al. 2018) and our findings extend this to WM manipulation performance. Furthermore, the PFC has been pointed out as especially age-sensitive to decline (Nyberg et al. 2010), and longitudinal research has revealed that those with maintained WM performance also maintained PFC activity, while those whose WM performance showed a decline showed decreases in PFC activity (Rieckmann et al. 2017; Nyberg et al. 2022). While our study lacks a long-term follow-up enabling conclusions regarding potential long-term brain maintenance, we would argue that this frontal upregulation paired with increased performance is a positive sign considering the otherwise age-related prefrontal decreases in activity accompanied by decline in performance.

We did not find a statistically significant between-group difference in change for either WM composite or for the in-scanner WM task; however, supramaximal HIT improved significantly in both WM composites. While it is hard to draw conclusions about the clinical significance of this change, the gains in WM performance for supramaximal HIT can be contrasted to longitudinal research finding a WM performance decline with age (Nyberg et al. 2020). This proposes a potential protective effect of supramaximal HIT, especially for those with subsequent gains in leg strength, boosting WM function and performance during a time in life when decline is what is expected. This is important, as WM decline is associated with decline in instrumental activities of daily living (Tomaszewski Farias et al. 2009; McDougall et al. 2019), and gains to leg strength are in turn important for maintaining functional performance in daily living and preventing falls (Tinetti and Kumar 2010).

While we did find a significant between-group difference in the right putamen, with supramaximal HIT decreasing activation and MIT increasing activation, this change was not related to a change in WM performance, VO_2_peak, or leg strength. The same is true for the area in the right cerebellum with a significant increase in activation irrespective of group. As neither area was related to WM performance, it is hard to interpret these functional changes. They could be related to other measurements; however, we have not explored this in the current study. Our limited findings with regard to between-group differences in functional change are not surprising, considering that the significant between-group difference in change for WM performance found in Simonsson et al. (2023) was not statistically significant for the MRI sample in this study. Furthermore, the intervention time itself was only 12 weeks, and it has been suggested that cognitive gains are primarily seen for longer interventions (Falck et al. 2019).

While the findings above are interesting, they are also based on a small sample size, and we were therefore unable to conduct rigorous corrections for multiple comparisons for our fMRI analyses. As such, these results are exploratory in nature and should be interpreted with caution. By using an extension threshold of 10 voxels, as well as an explicit mask on WM-related activity with a strict threshold, we have attempted to take precautionary measures to decrease the risk of spurious findings, but we acknowledge that these do not replace formal correction procedures. Accordingly, we emphasize that the reported effects do not meet corrected statistical thresholds and, until replicated in future studies, are best viewed as preliminary. A stricter correction for multiple comparisons would have been preferred (Poldrack et al. 2008). The design of the study is to identify between-group differences in change, and thus within-group change results, while interesting and promising, should be interpreted with caution before future studies can strengthen them. Future studies addressing individual differences in change to cognition may need to be dimensioned to increase variability in exercise-induced physiological, neural, and cognitive change. Furthermore, our study population, while nonexercising, was also relatively healthy and well educated, and studies on a wider range of older adults, including those with common diseases and/or lower socioeconomic status, would be of interest. This would help the field better understand how the mechanisms underlying exercise-induced WM change may differ depending on physical, neurological, and psychological function, as well as the influence of societal circumstances. For example, there is currently ongoing research on a similar supramaximal HIT protocol for patients with chronic obstructive pulmonary disease (Jakobsson et al. 2024), and we look forward to seeing whether mechanisms of cognitive change can be similarly identified in that population.

Despite the limitations, this was a rigorous RCT with both participants and outcome assessors blinded to group allocation during both physiological and cognitive testing. This study used gold-standard measurements of physiological adaptations, as well as an extensive battery of cognitive tests with multiple tests tapping different cognitive domains. Furthermore, the in-scanner task used has been extensively studied in longitudinal research and is considered reliable for the age group (Rieckmann et al. 2017). Models on exercise-induced cognitive change propose that physiological changes, such as changes to cardiorespiratory fitness, predict changes to brain structure and function, which in turn predict gains in cognitive function (Voss and Jain 2022). Our finding that leg strength gains may be a predictor for functional and performance change to WM memory following supramaximal HIT suggests yet another physiological mechanism that may need to be specifically targeted when prescribing exercise for cognitive improvements. Finding further active ingredients to cognitive gains is important to better understand who benefits cognitively from exercise, and why.

## Conclusions

For non-exercising older adults, supramaximal HIT led to superior effects in WM compared to MIT. Improved leg strength in the HIT group predicted upregulation of the dlPFC during a WM task, which in turn predicted increased WM performance. These results indicate that leg strength may be an important physiological target when prescribing exercise for cognitive improvements.

## Supplementary Material

supplementary_materials_bhaf277

## Data Availability

The data are available upon request from the corresponding author.
