# Supplementary material for: Working memory improvements following supramaximal high-intensity interval training predicted by increased prefrontal cortex activation and leg strength gains
Source: Cereb Cortex. 2025 Oct 14;35(10):bhaf277. doi: 10.1093/cercor/bhaf277 (PMC12526879; doi:10.1093/cercor/bhaf277)
Supplement: supplementary_materials_bhaf277 [file supplementary_materials_bhaf277.docx]

**Supplementary Materials**

Working memory improvements following supramaximal HIT predicted by increased prefrontal activation and leg strength gains

Authors: Sofi Sandström^a,b^*, Emma Simonsson^b,c^, Mattias Hedlund^c^, Erik Rosendahl^c^, & Carl-Johan Boraxbekk^a,b,d,e^.

Affiliations:

^a^Department of Diagnostics and Intervention, Umeå University, Umeå, Sweden.

^b^Umeå Center for Functional Brain Imaging, Umeå University, Umeå, Sweden.

^c^Department of Community Medicine and Rehabilitation, Umeå University, Umeå, Sweden.

^d^ Institute for Clinical Medicine, Faculty of Medical and Health Sciences, University of Copenhagen, Copenhagen, Denmark

^e^Institute of Sports Medicine Copenhagen (ISMC) and Department of Neurology, Copenhagen University Hospital Bispebjerg, Copenhagen, Denmark

*Corresponding author: Sofi Sandström, Umeå University 90187 Umeå, [sofi.sandstrom@umu.se](mailto:sofi.sandstrom@umu.se)

**Table S1.** *Within-group change and Between-group differences in change for all WM tasks in MRI sample.*

|  | Within-group change | | |  | | | Between-group difference in change | | | |
| --- | --- | --- | --- | --- | --- | --- | --- | --- | --- | --- |
|  | HIT | | | MIT | | | Group x Time | | | |
|  | No. | Mean | SE | No. | Mean | SE | Mean | 95% CI | *p* | ES |
| AOS | 22/20 | 2.09 | 1.58 | 19/20 | 0.19 | 1.66 | 1.90 | [6.44, -2.53] | 0.41 | 0.27 |
| BDS | 22/20 | 0.52* | 9.26 | 20/20 | 0.09 | 0.26 | 0.44 | [1.17, -0.26] | 0.24 | 0.39 |

*Caption: Values from linear mixed-effects models with individual as a random effect. Models are adjusted for age, sex, and years of education as fixed effects. Data are reported as LMM-estimated mean change with SE for within-group change, and 95% CI for between-group differences in change. ES was calculated from the model estimates of between-group difference in mean change and the unadjusted pooled standard deviation. HIT = Supramaximal High-Intensity interval Training; MIT = Moderate Intensity Training; No. = number of available measurements at baseline and follow-up presented as baseline/follow-up; CI = Confidence Interval; ES = effect size; * = significant within-group change (p<0.05) based on LMM estimates.*
